# Metabolomic and transcriptomic profiling reveals the effect of dietary protein and lipid levels on growth performance in loach (*Paramisgurnus dabryanus*)

**DOI:** 10.3389/fimmu.2023.1236812

**Published:** 2023-08-01

**Authors:** Zi-Rui Wang, Shu-Yao Li, Ya-Zhou Zhang, Yong-An Li, Huan-Huan Huo, Chuan-Qi Yu, Qiu-Bai Zhou

**Affiliations:** ^1^ College of Animal Science and Technology, Jiangxi Agricultural University, Nanchang, China; ^2^ Key Laboratory of Featured Hydrobios Nutritional Physiology and Healthy Breeding, Nanchang, China

**Keywords:** *Paramisgurnus dabryanus*, growth performance, protein, lipid, omics

## Abstract

The subject of this study was to explore the optimum requirements of loach (*Paramisgurnus dabryanus*) regarding dietary proteins and lipids and discuss the underlying mechanism. We designed nine diets to determine the effects of different levels of dietary crude protein (CP: 30%, 35%, and 40%) and ether extract (EE: 6%, 10%, and 14%) on the growth performance and metabolism of *P. dabryanus*. In total, 2160 healthy *P. dabryanus* (5.19 ± 0.01 g) were divided into nine groups with four replications at 60 fish per barrel stocking density. The trial lasted for eight weeks. Serum and liver samples were gathered for metabolomic and transcriptomic analyses. The results showed that the specific growth rate of *P. dabryanus* in the CP40EE10 group was the fastest and notably higher than that in other groups (*P<* 0.05). Analysis of the metabolome results found that the mTOR signaling pathway, glycerophospholipid metabolism, D-arginine and D-ornithine metabolism were significantly enriched pathways in the CP40EE10 group compared with the other groups (*P<* 0.05). Moreover, the transcriptomic analysis of differentially expressed genes (DEGs) showed that the expression of *ARG* (arginase) involved in protein synthesis was significantly upregulated in the CP40EE10 group compared to the slowest growing group (*P*< 0.05). Additionally, the expression of *SPLA2* (secretory phospholipase A2) involved in lipid metabolism and *FBP* (fructose-1,6-bisphosphatase) involved in glucose metabolism were all significantly downregulated in the CP30EE6 group compared with the CP40EE10 group (*P*< 0.05). Furthermore, the analysis of differentially expressed metabolites (DEMs) and DEGs co-enriched in the KEGG pathway revealed that the significantly enriched pathways were arginine and proline metabolism, glycerophospholipid metabolism, and glycolysis/gluconeogenesis in CP40EE10 compared with other groups (*P*< 0.05). We conclude that including 40% CP and 10% EE in the *P. dabryanus* diet could result in a better growth rate. We hypothesized from metabolomic and transcriptomic analyses that the CP40EE10 diet might promote the growth of *P. dabryanus* by promoting protein synthesis, lipid metabolism, and energy production.

## Introduction

*Paramisgurnus dabryanus* (*P. dabryanus*), a member of Cypriniformes, Cobitidae, *Paramisgurnus*, it is famous for its high survival rate, strong disease resistance and fast-growing (1). Moreover, the fillet of *P. dabryanus* richly contains high-quality protein, fatty acid, various mineral elements, and B vitamins, which are popular among consumers (2, 3). The nutritional value of wild loach is higher than that of farmed loach, but the yield of wild loach cannot meet the needs of consumers. Thus, an artificial culture of loach is necessary. At present, studies of loach have focused on investigating breeding and reproduction (4), hybridization (5), and immune responses (6, 7). However, research progress on the nutritional requirements of artificial feed for loach is slower than that of other fish (2, 8).

The suitable nutrient composition of feed ensures a nutritional balance to promote growth and minimizes production costs and nitrogen emissions. Furthermore, it has been reported that dietary lipids have a protein-retaining effect, and the addition of more lipids in the diet improves the efficient utilization of protein, thereby maximizing nitrogen retention and improving growth performance (9–11). As a result, evaluate and determine the optimal levels of dietary protein and lipid for the artificial diet of *P. dabryanus* is of great significance

Previous studies on the nutrient composition of the feed of aquatic animals tend to focus on apparent indicators, such as weight gain rate, survival rate, and biochemical blood indicators (11–13). However, more and more researchers have paid attention to understanding the growth of fish on the metabolic and molecular levels after technological developments (14–16). In recent years, the rapid development of integrated multi-omics analysis reported has provided new understanding for revealing the hidden biological regularities of various phenotypes (17–20). Transcriptomic data can be used to explore functional genes associated with phenotypes, but do not reflect actual metabolite level in an organism at the level of gene expression, which makes it hard to determine the critical path of specific character (21–23).

Metabolomics can be used to understand the physiological and biochemical states of biological systems about phenotypes at the metabolite level (24, 25). Therefore, an integrated analysis of metabolomics and transcriptomics can further link genes and metabolites to systematically characterize metabolic pathways associated with phenotypes (19, 26).

In previous studies, dietary protein requirements of *Paramisgurnus dabryanus* (3 to 6 g) were from 33% to 36% and lipid levels were from 5% to 7% to achieve optimal growth performance (1, 27–29). Thus, 3 protein levels of 30%, 35%, 40% and 3 lipid levels of 6%, 10%, 14% were designed to evaluate the interaction effects of different dietary protein and lipid levels on growth performance and investigated the optimal balance of protein and lipid in *P. dabryanus* diets and the mechanisms behind it using LC-MS/MS metabolomics approach and the RNA-seq transcriptomics approach. These findings will strengthen our comprehension of the mechanisms behind growth differences and help to explore optimal nutritional requirements and feeding regimens of *P. dabryanus*.

## Materials and methods

### Experimental diets, animal and design

Soybean meal and fish meal were the main sources of protein, soy lecithin, soybean oil and fish oil were the main sources of lipid. A complete crossover experiment with three levels of crude protein (CP) and three levels of ether extract (EE) was used to formulate a total of 9 diets with four replicates (**Table 1**). The ingredients were ground to an ideal particle size (80 mesh), weighed, mixed and added water to stir evenly according to the formula, then used a granulator to form feed pellets with a diameter of 2 mm, which were dried for later use.

**Table 1:** The composition and nutrient level of experimental feed (air-dry basis) %.

Items	Groups								
	CP30EE6	CP30EE10	CP30EE14	CP35EE6	CP35EE10	CP35EE14	CP40EE6	CP40EE10	CP40EE14
Ingredients									
Fish meal	20.00	20.00	20.00	20.00	20.00	20.00	20.00	20.00	20.00
Full-fat expanded soybean	5.00	10.00	15.00	5.00	10.00	15.00	5.00	10.00	15.00
Soybean meal	22.50	19.00	15.50	22.50	19.00	15.50	22.50	19.00	15.50
Rapeseed meal	5.00	5.00	5.00	5.00	5.00	5.00	5.00	5.00	5.00
Wheat flour	8.00	8.00	8.00	8.00	8.00	8.00	8.00	8.00	8.00
Corn meal	20.00	20.00	20.00	20.00	17.60	12.90	15.00	10.10	5.40
Soybean oil: Fish oil (1:1)	2.00	5.40	8.60	2.00	5.40	8.60	2.00	5.40	8.60
Soy protein concentrate	0.00	0.00	0.00	7.50	7.50	7.50	15.00	15.00	15.00
Cellulose	11.0	6.10	1.40	3.50	1.00	1.00	1.00	1.00	1.00
Sodium carboxymethyl cellulose	2.00	2.00	2.00	2.00	2.00	2.00	2.00	2.00	2.00
Ca(H_2_PO_4_)_2_	2.00	2.00	2.00	2.00	2.00	2.00	2.00	2.00	2.00
Premix^1^	1.00	1.00	1.00	1.00	1.00	1.00	1.00	1.00	1.00
Soy lecithin	1.00	1.00	1.00	1.00	1.00	1.00	1.00	1.00	1.00
Choline chloride (50%)	0.50	0.50	0.50	0.50	0.50	0.50	0.50	0.50	0.50
Nutrients level^2^, %									
Moisture	9.51	9.36	8.80	9.77	9.41	8.62	9.38	9.12	8.92
CP	29.81	29.90	29.77	34.80	34.22	35.53	39.10	39.98	40.54
EE	3.56	8.21	12.07	3.63	8.38	12.67	4.85	9.22	14.24
Gross energy (kJ/g)^3^	14.63	16.49	18.28	15.88	17.34	18.32	16.26	17.28	18.26
Ash	7.18	7.18	7.19	7.50	7.64	8.45	8.33	8.74	8.76

^1^ The premix provided the followings per kg of diet: VA 5,000 IU, VB_1_ 25 mg, VB_2_ 45 mg, VB_6_ 20 mg, VB_12_ 0.1 mg, VK_3_ 10 mg, VE 200 mg, VC 200 mg, VD_3_ 2,500 IU, inositol 200 mg, pantothenic acid 60 mg, niacin 200 mg, folic acid 10 mg, biotin 1.5 mg, NaSeO_3_·5H_2_O 0.3 mg, CoCl_2_·6H_2_O 0.4 mg, KI 0.8 mg, CuSO_4_·5H_2_O 10 mg, MnSO_4_·4H_2_O 20 mg, ZnSO_4_·H_2_O 50 mg, FeSO_4_·7H_2_O 150 mg, MgSO_4_·7H_2_O 500 mg, NaCl 1,000 mg.

^2^ Nutrient levels were measured values.

^3^Calculated using the mean values for carbohydrates (17.2 kJ/g), proteins (23.6 kJ/g), and lipids (39.5 kJ/g) according to NRC (2011).

*Paramisgurnus dabryanus* conducted in the sunshine shed of the breeding base of Jiangxi Agricultural University were from the Fengcheng Loach Breeding Professional Cooperative of Jiangxi province, China. In this study, a total of 2160 healthy *P. dabryanus* (5.19 ± 0.01 g) were divided into nine groups with four replications at a stocking density of 60 fish per tank (80 cm × 66 cm × 64 cm), and fed with nine diets (**Table 1**) twice-daily (08:00 and 17:00) for an eight-week period according to 3% of the fish body weight. During the trial, 1/2 water was exchanged once a week with continuous aeration throughout the experimental period. The water temperature was 25~28°C and the dissolved oxygen was above 5 mg/L, and the photoperiod was determined by the natural lighting. All experimental procedures were performed according to the regulations in the China Law for Animal Health Protection and Instructions (Ethics approval No. SCXK (YU2005-0001)).

### Sample collection

At the end of the feeding experiment, the number and weight of loaches were obtained after 24 hours of starvation to evaluate growth performance. Four loaches per barrel were anesthetized with MS-222 (100 mg/mL). Then wipe the surface of the loaches with 75% ethanol. Blood samples of 8 loaches from each group were collected, centrifuged at 3000 r/min for 10 min, and serum samples were collected for metabolomics analysis. Liver samples of 8 loaches form each group were immediately stored in liquid nitrogen for liver transcriptome analysis. All samples were transferred to an ultra-low-temperature refrigerator at −80 °C.

All chemical composition analyses of diets were conducted according to the methods specified by the AOAC (1995). Moisture was analyzed by 105 °C normal temperature drying method (GB/T 6435-2014), crude protein (N × 6.25) by Kjeldahl method (GB/T 6432-1994), crude lipid by Soxhlet extraction method (GB/T 6433-2006) and ash analysis by combustion at 550°C using a muffle furnace (GB/T6438-2007).

### Performance measurement


WGR(weight gain ratio,%)=100×(FBW–IBW)/IBW;

SGR(specific growth rate,%/d)=100×[Ln(FBW)–Ln(IBW)]/d

IBW is the initial body weight (g), FBW is the final body weight (g), d denotes days of feeding.

### Metabolomic assay

Metabolomic was performed in OE Biotech Co. Ltd., Shanghai, China. The sample was added into 1.5 mL EP tube with 10 μL of 2-chloro-l-phenylalanine (0.3 mg/mL) dissolved in methanol as the internal standard. After 10 seconds of vorticity, 300 μL of ice-cold mixture of methanol and acetonitrile (2:1) was added, and vortexed for 1 minute, then it is ultra sounded on ice for 10 minutes and incubation at −20°C for 30 minutes to precipitated proteins. The extract was centrifuged at 13000 rpm for 10 min at 4 °C, and the supernatant was taken from a 300 µL glass vial and dried by freezing and concentrating in a centrifugal dryer. A mixture of 400 µL methanol and water (1:4) were added to each sample, the sample was swirled for 30 seconds, ultra sounded for 3 minutes, and then placed at -20 °C for 2 hours. The sample was centrifuged at 13000 rpm for 10 min at 4 °C. Each tube of supernatants (150 µL) was collected with a crystal syringe, filtered through a 0.22 µm microfilter, and transferred into LC vials. The samples were stored at −80°C for LC-MS analysis. Quality control (QC) samples were prepared by mixing equal portions of all samples as a single combined sample (30).

ACQUITY UPLC I-Class system (Waters Corporation, Milford, USA) was used in conjunction with VION IMS QTOF mass spectrometry to analyze ESI positive ion and ESI negative ion mode metabolic profiles. ACQUITY UPLC BEH C18 column (100×2.1 mm, 1.7 µm) was used for both modes. The column temperature was 45°C. Water containing 0.1% formic acid and acetonitrile/methanol 2/3 were used as mobile phases A and B, respectively. The gradient program was 0/99, 1/70, 2.5/40, 6.5/10, 8.5/0, 10.7/0, 10.8/99, 13/99 (min/% mobile phase A), and the flow rate of mobile phase was 0.4 mL/min. All samples were kept at 4 °C during analysis. A mass spectrometry (MS) system equipped with ESI source was used to scan samples with a mass range of 50 to 1000 in both positive and negative ion modes. The parameters of MS were as follows: electrospray capillary voltage 2500 V, injection voltage 40 V, collision voltage 4 eV, ion source temperature 115°C, desolving temperature 450 m, desolving gas flow 900 L/h (30).

The data was preprocessed prior to pattern recognition, and the raw data was subjected to baseline filtering, peak recognition, integration, retention time correction, peak alignment, and normalization by metabolomics software Progenesis QI V2.3 (Nonlinear Dynamics, Newcastle, UK). Human Metabolome Database (HMDB), Lipidmaps (V2.3), METLIN database and self-built database were used to identify compounds based on accurate mass numbers, secondary fragments and isotope distribution. Differential metabolites were screened by partial least squares discriminant analysis (PLS-DA) model with projected variable importance (VIP≥1), and T-test results were obtained by univariate analysis (*P*< 0.05).

### Transcriptomic assay

Transcriptomics was performed in OE Biotech Co. Ltd., Shanghai, China. Total RNA was isolated using the Mir-VANA miRNA kit (Cat. AM1561, Invitrogen, Thermo Fisher Scientific Inc., USA), following manufacturer’s protocol. RNA purity and quantification were assessed using a NanoDrop 2000 spectrophotometer (Thermo Fisher Scientific, Waltham, MA, USA). RNA integrity was assessed using the Agilent 2100 bioanalyzer (Agilent Technologies, Santa Clara, CA, USA). The library was then constructed using the TruSeq stranded mRNA LT sample preparation kit (Illumina, San Diego, CA, USA) according to the manufacturer’s instructions.

High-quality reads were screened by removing all reads containing adapters, containing > 10% unknown nucleotides and > 50% low-quality bases. Then, *De novo* splicing was conducted. Trinity software was used to obtain the transcription sequences by pairing end splicing (31). According to sequence similarity and length, the longest one was selected, and the CD-HIT software was used for clustering to eliminate redundancy, and a set of final Unigene was obtained as a reference sequence for subsequent analysis (32). BUSCO has constructed single-copy gene sets for several extensive evolutionary clays from the OrthoDB database (33). Unigene was compared with NR, KOG, GO, Swiss-Prot, eggNOG and KEGG databases by diamond software (34) and Pfam database by HMMER software (35). Using the spliced Unigene as a database, the expression abundance of Unigene in each sample was identified by sequence similarity comparison. Bowtie2 was used to calculate the number of reads compared with Unigene in each sample (36), and the expression level of Unigene (FPKM value) was calculated. These values were then compared to explore differences in gene expression between the samples. DESeq2 was used for inter-group and inter-sample comparisons to identify DEGs. KEGG pathway enrichment analysis was performed on these DEGs relative to the genome-wide background using the same method as the differential abundance metabolite analyses described above.

### Statistical analysis

The results of growth performance data were determined using one-way analysis of variance, followed by Duncan’s multiple comparisons test. Optimal nutrient combination levels were obtained by two-factor analysis of variance. The data were presented as mean ± SE; significant differences (*P*< 0.05) between variables. Statistical analysis was performed using SPSS 25.0.

## Results

### Growth performance

**Figure 1:**
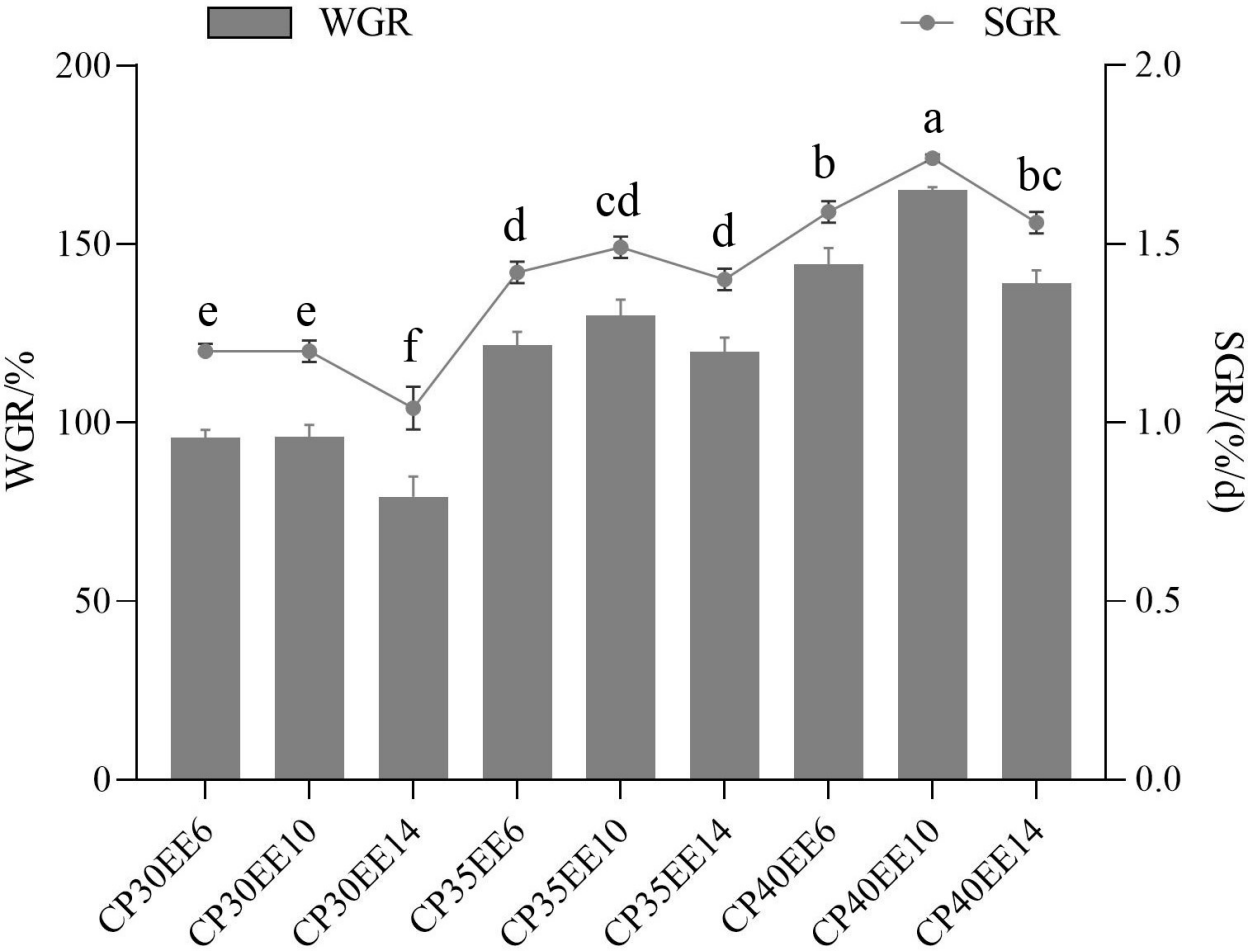
Effect of different combinations of lipid and protein levels in diets on growth performance of *Paramisgurnus dabryanus* WGR, weight gain rate; SGR, specific growth rate; Diets: CP30EE6: 30% crude protein, 6% ether extract; CP30EE10: 30% crude protein, 10% ether extract; CP30EE14: 30% crude protein, 14% ether extract; etc.; Treatment means represent the average values for 4 tanks per treatment; treatment means followed by different superscript letter in the same column are significantly different (*P *< 0.05).

The combination of different lipid and protein levels in the diet significantly affected the WGR and SGR of *P. dabryanus* (*P<* 0.05; **Figure 1**). The WGR and SGR of *P. dabryanus* in CP40EE10 were higher than those in the other groups. On the other hand, CP30EE6 as a low-protein/low-lipid diet group and CP30EE14 as a low-protein/high-lipid diet group were inferior to the CP40EE10 group. Next, a pairwise comparison of CP30EE6 vs. CP40EE10 and CP30EE14 vs. CP40EE10 were conducted to analyze the molecular mechanisms affecting the growth of *P. dabryanus*.

### Serum metabolome analysis

#### Multivariate statistical analysis

**Figure 2:**
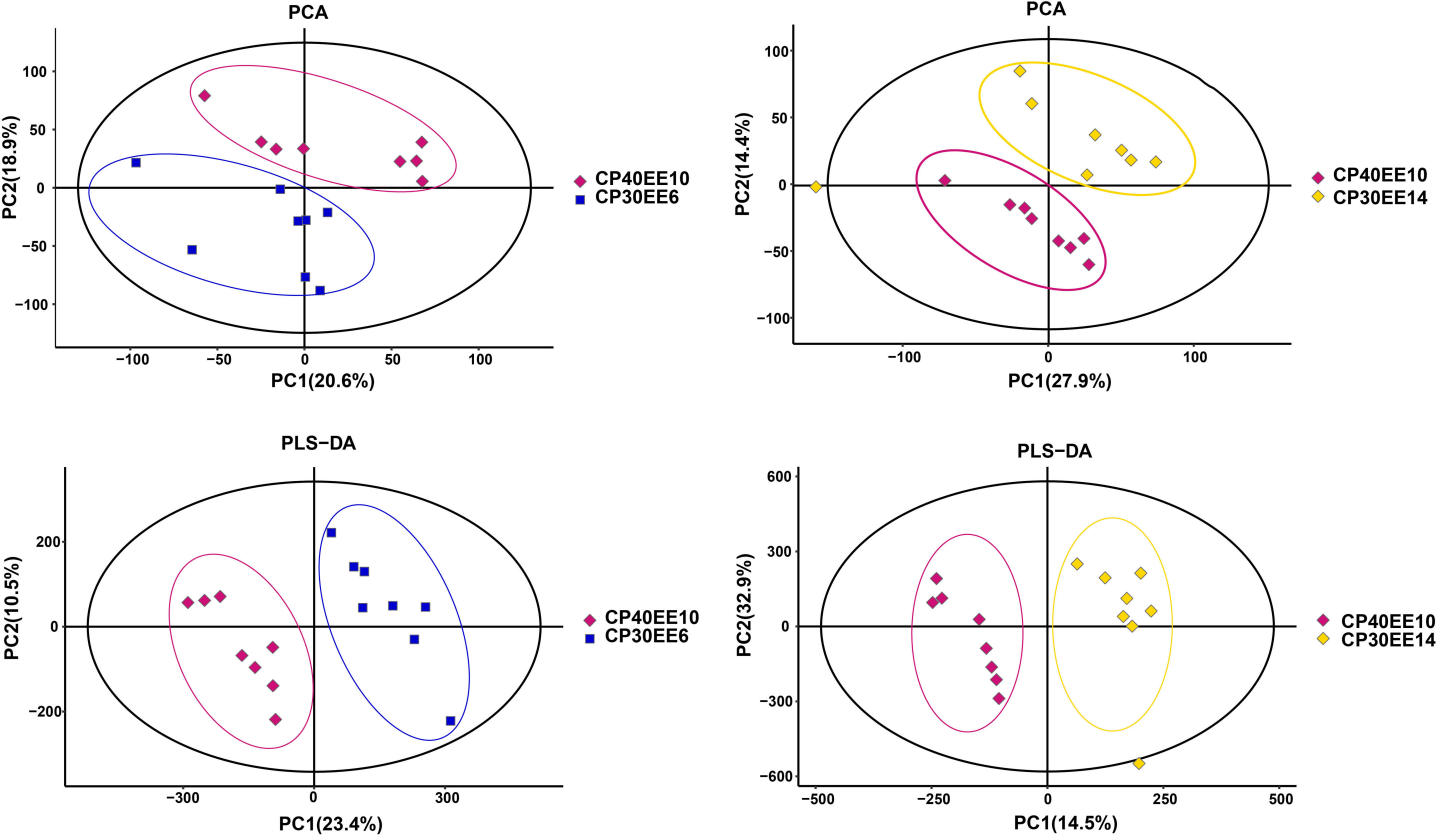
PCA and PLS-DA score plots of serum metabolites in samples. CP30EE6, 30% crude protein, 6% ether extract; CP30EE14, 30% crude protein, 14% ether extract; CP40EE10, 30% crude protein, 10% ether extract.

Principal component analysis (PCA) was used to reduce the dimensionality of the detected metabolite data to analyze the grouping trends and outliers of the observed variables in the dataset. There was a clear separation between the CP40EE10 vs. CP30EEE6 and CP40EE10 vs. CP30EE14 groups (**Figure 2**). Besides, PLS-DA score plots showed an apparent division between groups (**Figure 2**). Combining the above two results, the CP40EE10 group was significantly different from both the CP30EE6 and CP30EE14 groups regarding their metabolite levels (*P<* 0.05).

#### Differential metabolite screening

**Figure 3:**
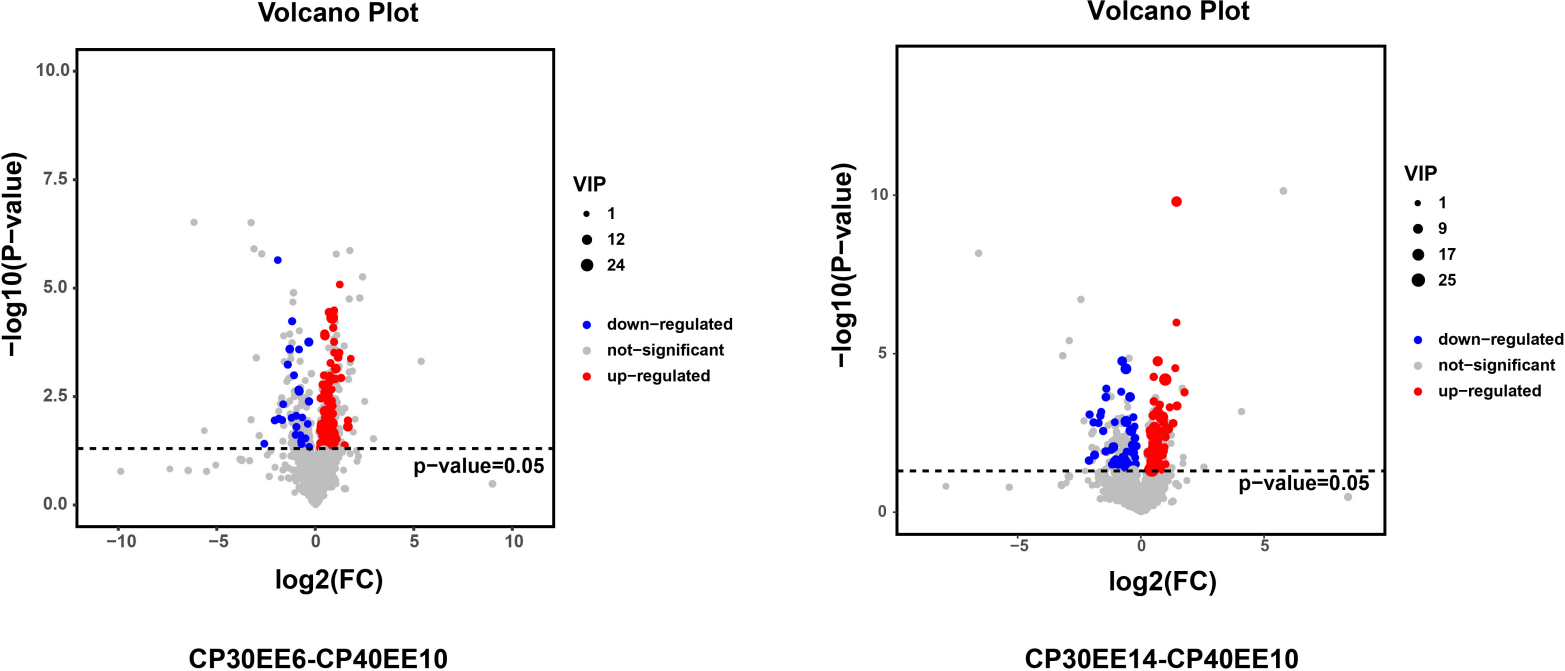
Volcanic plot of DEMs. DEMs, differentially expressed metabolites; CP30EE6, 30% crude protein, 6% ether extract; CP30EE14, 30% crude protein, 14% ether extract; CP40EE10, 30% crude protein, 10% ether extract.

**Figure 4:**
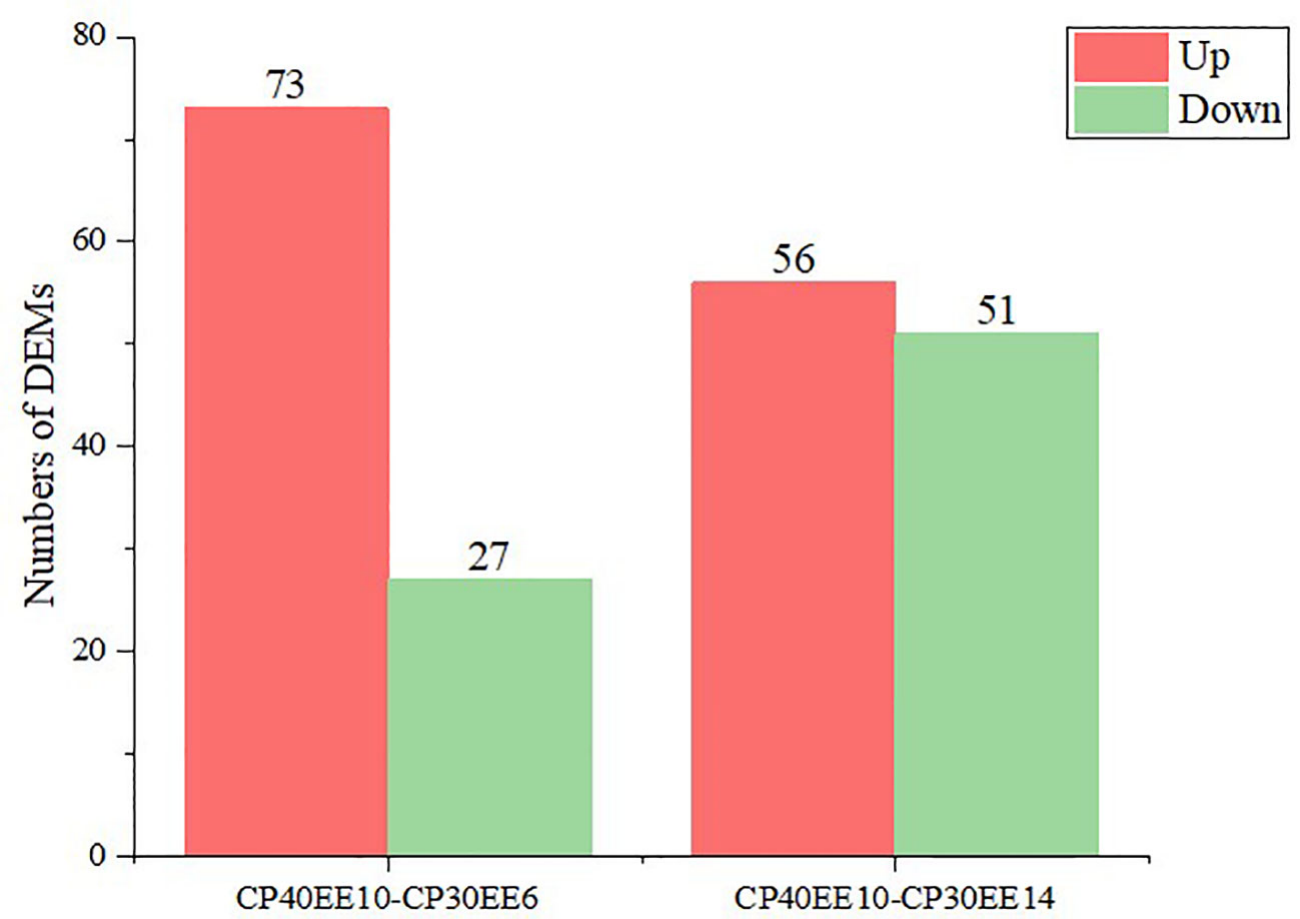
Statistics of DEMs in CP40EE10 vs CP30EE6 and CP40EE10 vs CP30EE14. Red represents significant up-regulation, and green represents significant down-regulation (Same as below). DEMs, differentially expressed metabolites; CP30EE6, 30% crude protein, 6% ether extract; CP30EE14, 30% crude protein, 14% ether extract; CP40EE10, 30% crude protein, 10% ether extract.

LC-MS/MS was used to analyze the serum of eight replicate samples from CP30EE6, CP30EE14, and CP40EE10. A total of 1492 metabolites were detected. There were 100 differentially expressed metabolites (DEMs) in the CP40EE10 group compared with the CP30EE6 group, including 73 metabolites markedly upregulated and 27 metabolites significantly down-regulated (*P<* 0.05). Meanwhile, there were a total of 107 DEMs in the CP40EE10 group, with 56 metabolites obviously upregulated and 51 metabolites obviously downregulated (*P<* 0.05) (**Figures 3**, **4**), compared with the CP30EE14 group.

#### Enrichment analysis of metabolic pathways

**Figure 5:**
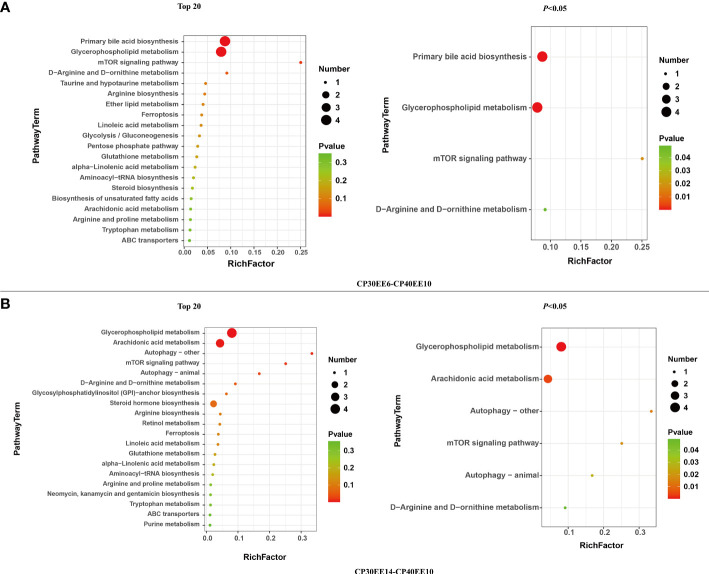
Metabolome view map of significant metabolic pathways. DEMs, differentially expressed metabolites; CP30EE6, 30% crude protein, 6% ether extract; CP30EE14, 30% crude protein, 14% ether extract; CP40EE10, 30% crude protein, 10% ether extract. The X-axis means rich factor. The Y-axis represents the KEGG pathway terms. The roundness color represents the p value. The roundness area represents the DEM number in this pathway. **(A, B)** represent differential metabolic pathway comparison between CP30EE6-CP40EE10 and CP30EE14-CP40EE10, respectively

**Figure 6:**
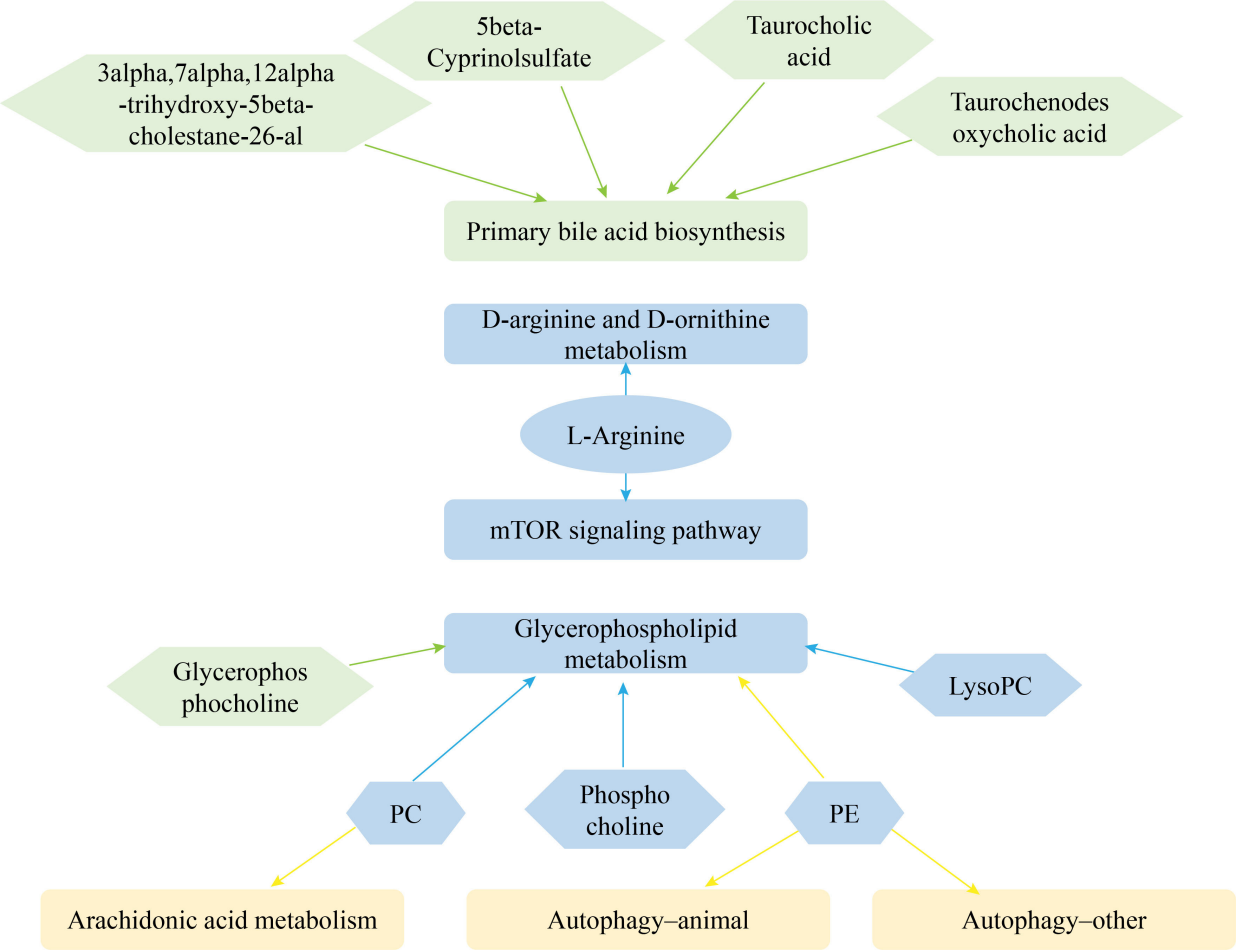
Network of metabolic pathways and key DEMs. Green represents DEMs and significantly enriched pathways in CP40EE10 vs CP30EE6 groups, yellow represents DEMs and significantly enriched pathways in CP40EE10 vs CP30EE14, and blue represents DEMs and significantly enriched pathways in common.

A total of 20 and 21 metabolic pathways were detected in CP40EE10 vs. CP30EE6 and CP40EE10 vs. CP30EE14, respectively (**Figure 5**), in the enrichment analysis of the above DEMs in the KEGG pathway. Primary bile acid biosynthesis, mTOR signaling pathway, glycerophospholipid metabolism, D-arginine and D-ornithine metabolisms were the first four significant differential pathways between the CP40EE10 and CP30EE6 groups (**Figure 5A**). The glycerophospholipid metabolism, arachidonic acid metabolism, autophagy–other, mTOR signaling pathway, autophagy–animal, and D-arginine and D-ornithine metabolisms were the first six significant differential pathways between the CP40EE10 and CP30EE14 groups (**Figure 5B**). The three metabolic pathways, glycerophospholipid metabolism, mTOR signaling pathway, and D-arginine and D-ornithine metabolisms in the CP40EE10 group were significantly enriched compared with the other two groups (*P<* 0.05), as shown in **Figure 6**. In these pathways, L-arginine was available in the mTOR signaling pathway and D-arginine and D-ornithine metabolism. Phosphatidylcholine (PC), 1-Acyl-sn-glycero-3-phosphocholine (LysoPC), glycerophosphocholine, phosphocholine, and phosphatidylethanolamine (PE) were involved in glycerophospholipid metabolism. The expression of these DEMs in the CP40EE10 group was significantly higher than in the other two groups.

### Liver transcriptome analysis

#### Transcriptome sequencing and *De novo* assembly

Performing RNA sequencing on RNA samples extracted from liver tissues of the CP30EE6, CP30EE14, and CP40EE10 groups, transcriptomic sequencing of 24 samples was completed in this analysis, and 83.82 G clean bases were obtained. The valid base amount of each sample ranged from 94.26% to 95.40%, the Q30 base ranged from 95.41% to 95.85%, and the average GC content was 46.44%. After filtering, 57,001 unigenes were assembled, with a total length of 62,346,987 bp and an average length of 1,093.79 bp (**Supplementary Table S1**). Sequences ranging from 301 to 400 bp in length were the most abundant, constituting 24.93% of the total sequences (**Figure S1A**).

Transcriptional integrity can be estimated from conserved genes in related species. The total number of genes in the selected BUSCO groups was 4584, and 63.1% of the genes were encoded as complete proteins. Among these genes, 58.3% were complete. Among single-copy BUSCOs, 4.8% of the genes were complete and duplicated BUSCOs, 9.3% were fragmented BUSCOs, and 27.6% were missing BUSCOs (**Figure S1B**). The data were stored in the NCBI Sequence Read Archive (SRA, http://www.ncbi.nlm.nih.gov/Traces/sra) with accession number SRP384751.

#### Unigene expression level analysis

The assembled single genes were used as the database, and the expression abundance of single genes in each sample was determined by sequence similarity comparison. Bowtie2 was used to obtain the number of reads in each sample compared to single genes (**Supplementary Table S2**) and could represent the entire sequencing results, meeting the transcriptome data analysis requirements.

#### Detection of DEGs

**Figure 7:**
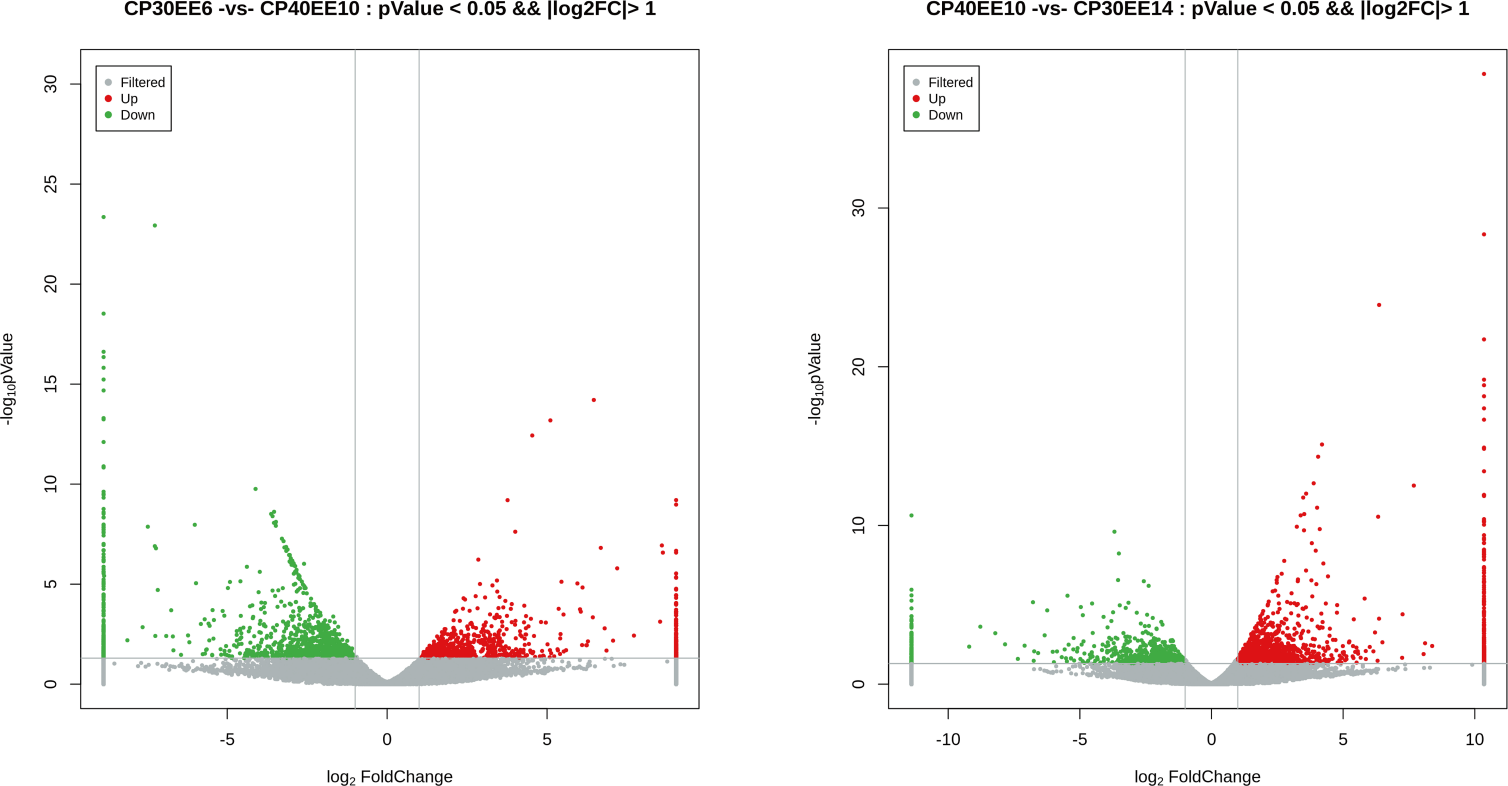
Volcanic plot of DEGs. DEGs, differentially expressed metabolites; CP30EE6, 30% crude protein, 6% ether extract; CP30EE14, 30% crude protein, 14% ether extract; CP40EE10, 30% crude protein, 10% ether extract.

**Figure 8:**
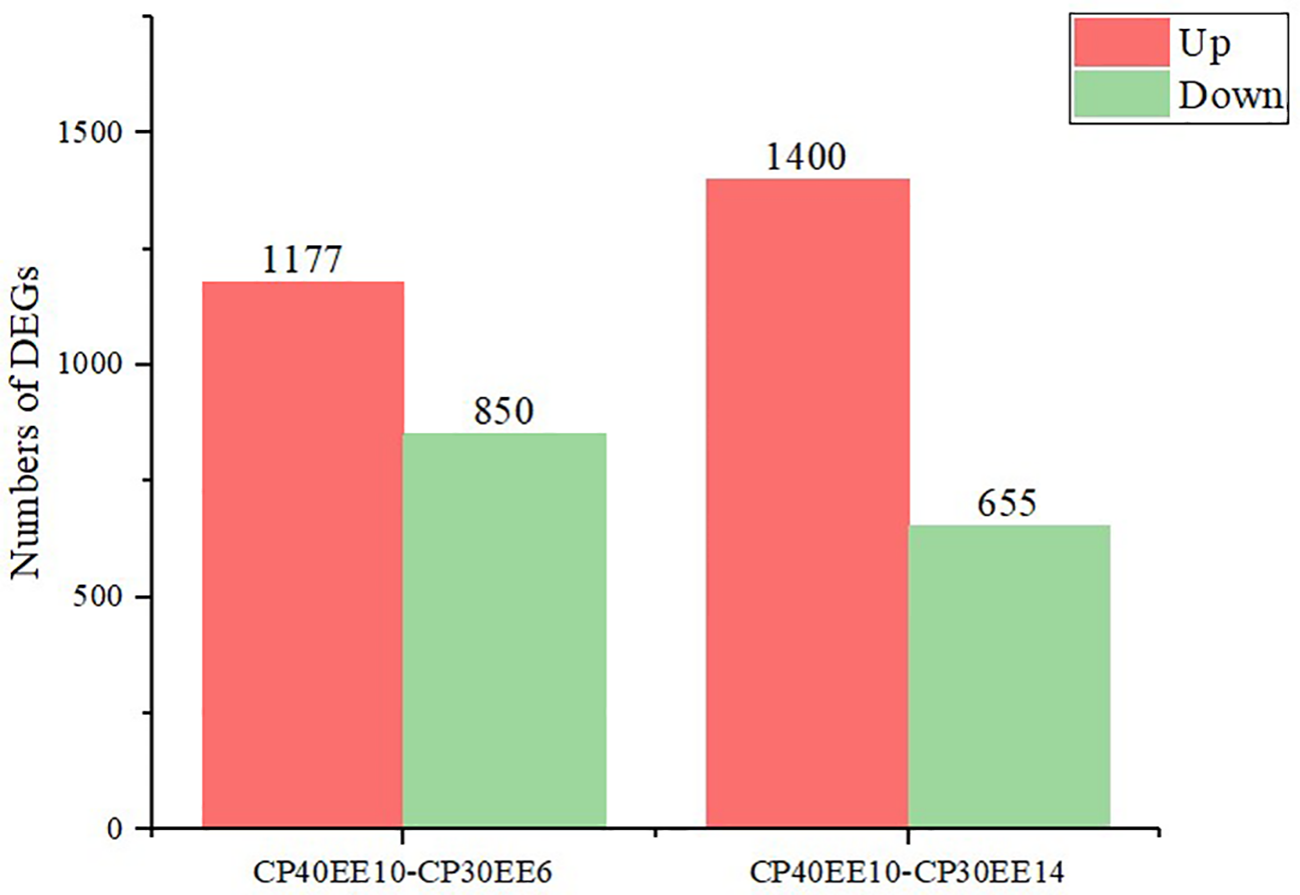
Statistics of DEGs in CP40EE10 vs CP30EE6 and CP40EE10 vs CP30EE14. DEGs, differentially expressed metabolites; CP30EE6, 30% crude protein, 6% ether extract; CP30EE14, 30% crude protein, 14% ether extract; CP40EE10, 30% crude protein, 10% ether extract.

This study used *P* ≤ 0.05 and FC ≥ 2 as the thresholds for significant differences in gene expression. Differentially expressed genes (DEGs) in the livers of *P. dabryanus* from the CP30EE6, CP30EE14, and CP40EE10 groups were identified. This approach identified 2027 and 2055 DEGs in CP40EE10 vs. CP30EE6 and CP40EE10 vs. CP30EE14, respectively. We found that 850 DEGs were significantly downregulated, while 1177 DEGs were significantly upregulated in the CP40EE10 group compared to CP30EE6. Simultaneously, compared with the CP30EE14 group, the CP40EE10 group had 1400 significantly upregulated DEGs, and 655 downregulated DEGs (**Figures 7**, **8**). Pathway analyses were conducted using the KEGG database to characterize the DEGs functionally. The CP30EE6 group had 180 downregulated DEGs distributed in 240 pathways compared with the CP40EE10 group. Among these pathways, 59 significantly enriched pathways were identified via KEGG pathway analysis (*P*< 0.05).

The CP30EE14 group had 242 downregulated DEGs distributed in 253 pathways. Among these pathways, there were 69 significantly enriched pathways (*P*< 0.05). In the CP40EE10 vs. CP30EE6 comparison, the 20 high-ranking significant pathways included seven metabolism-related pathways, such as arginine and proline metabolism, linoleic acid metabolism, alpha-linolenic acid metabolism, arachidonic acid metabolism, pentose phosphate pathway, ether lipid metabolism, and glycolysis/gluconeogenesis. The remaining 13 pathways were linked to digestion, disease, and immunity. Among them, three apparent pathways were pancreatic secretion, protein or fat digestion, and absorption (*P<* 0.05). In the CP40EE10 vs. CP30EE14 comparison, the 20 high-ranking significant pathways included 16 metabolism pathways, such as phenylalanine metabolism, glycolysis/gluconeogenesis, phenylalanine, tyrosine, and tryptophan biosynthesis.

**Figure 9:**
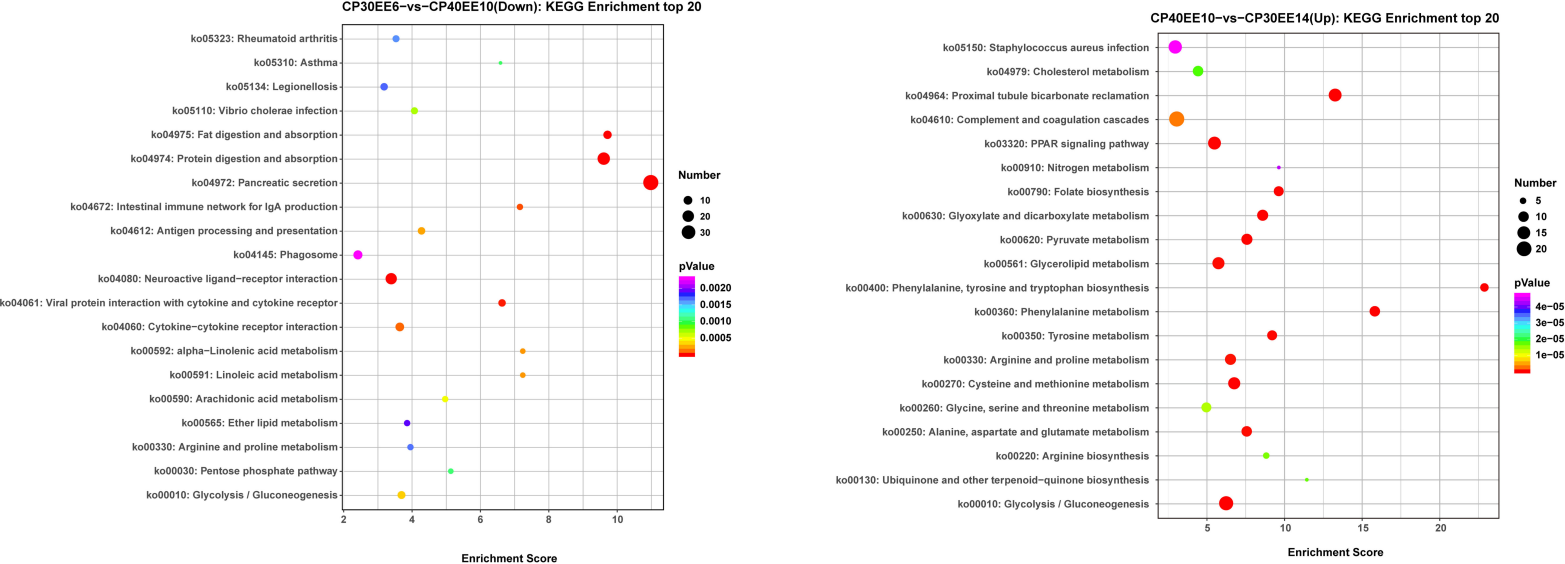
KEGG enrichment top 20 between CP30EE6, CP30EE14 and CP40EE10 groups. CP30EE6, 30% crude protein, 6% ether extract; CP30EE14, 30% crude protein, 14% ether extract; CP40EE10, 30% crude protein, 10% ether extract. The X-axis means rich factor. The Y-axis represents the KEGG pathway terms. The roundness color represents the p value. The roundness area represents the DEG number in this pathway.

**Figure 10:**
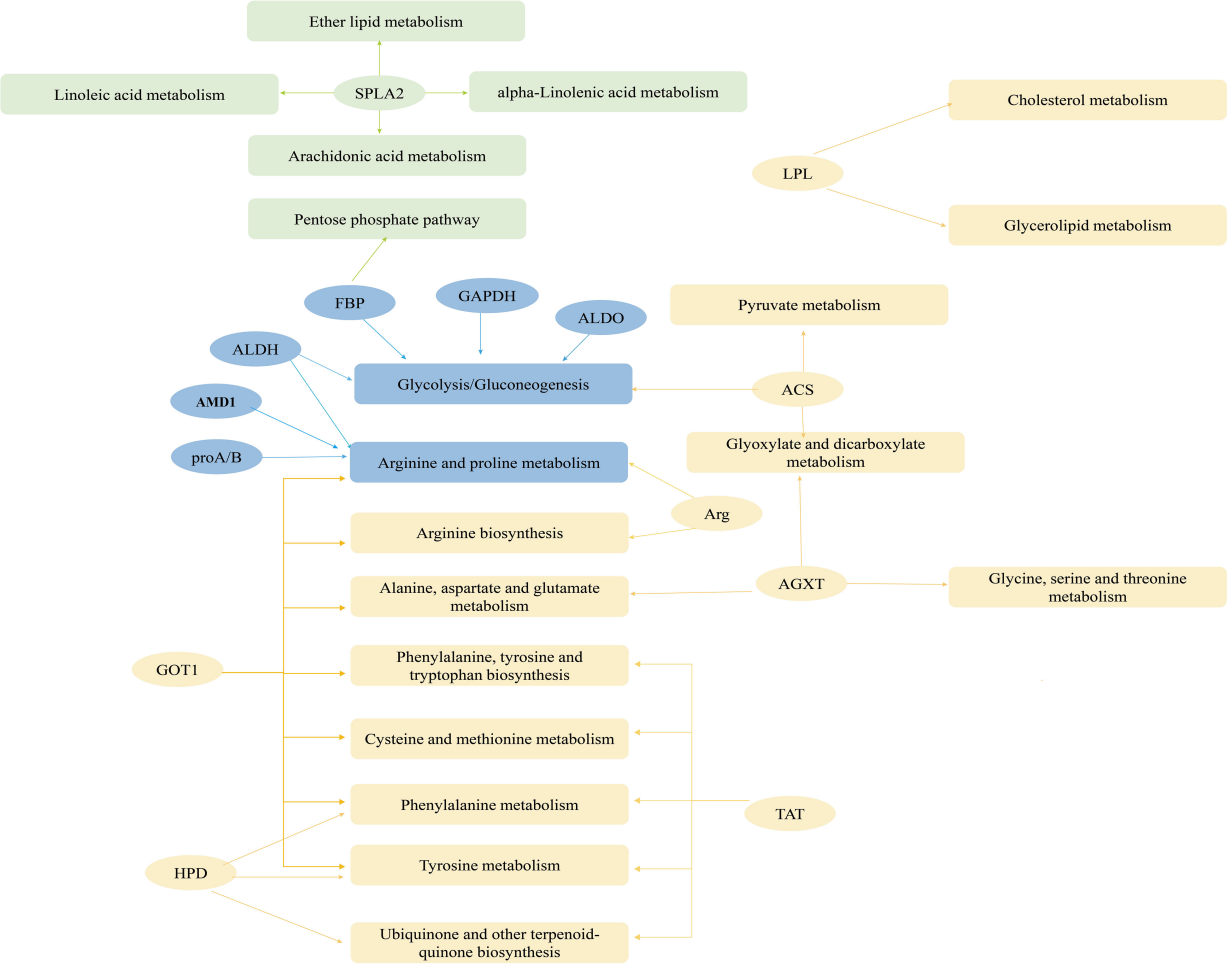
Network of metabolic pathways and key genes. Green represents DEGs and significantly enriched pathways in CP40EE10 vs CP30EE6 groups, yellow represents DEGs and significantly enriched pathways in CP40EE10 vs CP30EE14, and blue represents DEMs and significantly enriched pathways in common.

The PPAR signaling pathway and proximal tubule bicarbonate reclamation were enriched in the remaining four pathways (*P<* 0.05; **Figure 9**). PPAR contained all upregulated genes in seven metabolism-related pathways of the CP40EE10 vs. CP30EE6 comparison and 16 metabolism-related pathways of CP40EE10 vs. CP30EE14, as shown in **Supplementary Table S3**. Additionally, the main DEGs involved in glycolysis/gluconeogenesis and arginine and proline metabolism, such as *proA/B* (glutamate-5-semialdehyde dehydrogenase/glutamate 5-kinase), *FBP* (fructose-1,6-bisphosphatase), *GAPDH* (glyceraldehyde 3-phosphate dehydrogenase), *ALDO* (fructose-bisphosphate aldolase), and *AMD*1 (S-adenosylmethionine decarboxylase) in the CP40EE10 group, were higher than those in the CP30EE6 and CP30EE14 groups. The main metabolic pathways and key upregulated DEGs are shown in **Figure 10**.

Additionally, the DEGs significantly decreased in pancreatic secretion, protein or fat digestion, and absorption pathways in the CP30EE6 group were *amyA* (alpha-amylase), *PRSS*1-2-3 (trypsin), *LIPF* (gastric triacylglycerol lipase), *SPLA*2 (secretory phospholipase A2), and *NPC*1*L*1 (Niemann-Pick C1-like protein 1). Additionally, the DEGs significantly decreased in the proximal tubule bicarbonate reclamation. The PPAR signaling pathway in the CP30EE14 group were *PPARα* (peroxisome proliferator-activated receptor alpha), *gdhA* (glutamate dehydrogenase (NAD(P)+), *PCK* (phosphoenolpyruvate carboxykinase (GTP), *LPL* (lipoprotein lipase), and *ACS* (acyl-CoA synthetase) (**Supplementary Table S4**).

#### Combined analysis of DEMs and DEGs

**Figure 11:**
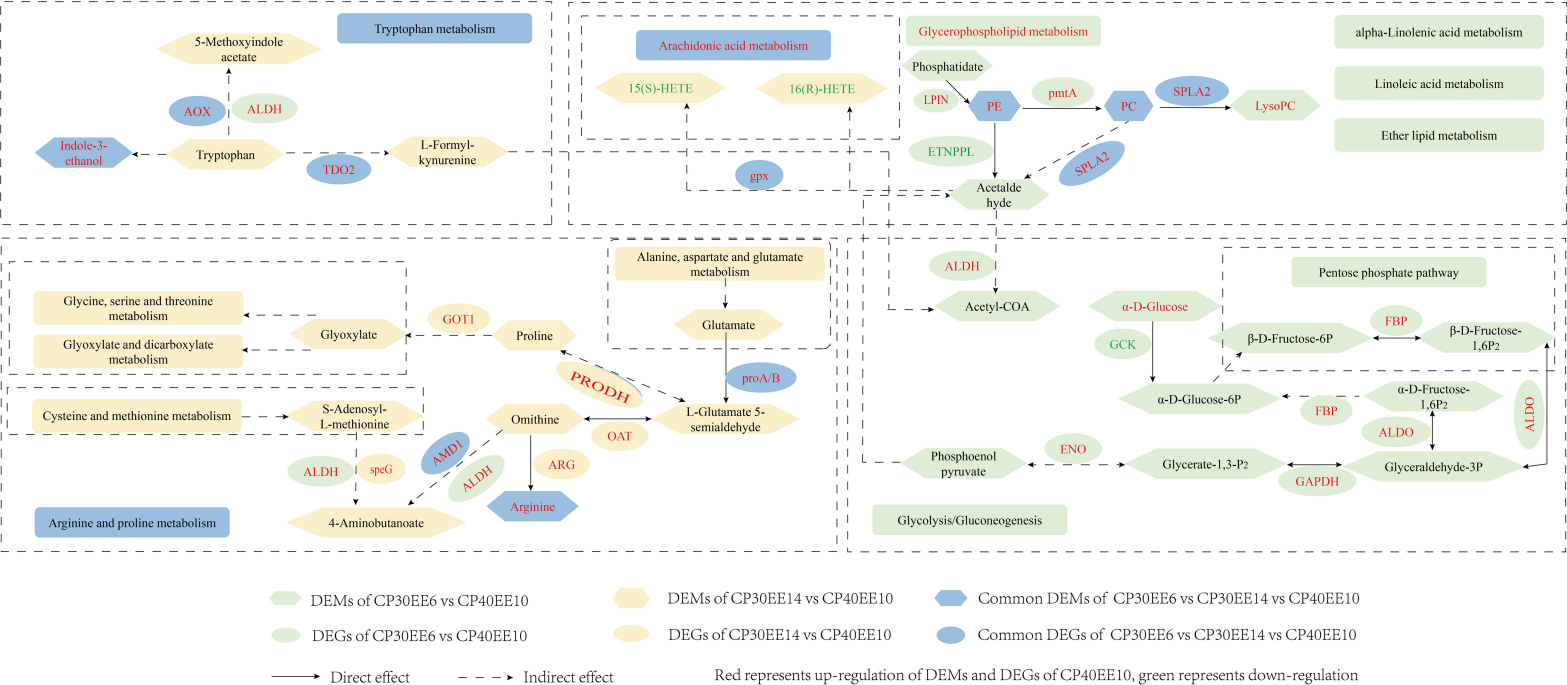
A metabolic network of DEMs and DEGs in KEGG pathways identified by multi-omics analysis between CP30EE6, CP30EE14 and CP40EE10 groups. DEMs, differentially expressed metabolites; DEGs, differentially expressed genes; CP30EE6, 30% crude protein, 6% ether extract; CP30EE14, 30% crude protein, 14% ether extract; CP40EE10, 30% crude protein, 10% ether extract.

The only significantly enriched metabolic pathway in the CP40EE10 vs. CP30EE6 comparison, in which DEMs and DEGs coexist, was glycerophospholipid metabolism (indicated by rounded green rectangles, *P<* 0.05). Only arachidonic acid metabolism and autophagy were significantly enriched pathways for DEM and DEG coexistence in the CP40EE10 vs. CP30EE14 comparison (indicated by rounded yellow rectangles, *P<* 0.05). Arachidonic acid, arginine and proline, and tryptophan metabolisms coexisted in the two comparison groups (indicated by rounded blue rectangles). Several critical metabolic pathways are shown in **Figure 11**. The metabolites and genes related to the amino acid, lipid, and carbohydrate metabolism in the serum and liver of *P. dabryanus* in the CP40EE10 group were significantly higher than those in the other two groups (*P<* 0.05).

## Discussion

Research on suitable feed formulations is essential for the aquaculture industry. Inappropriate protein and lipid levels lead to poor fish growth (13, 37–40). Therefore, by feeding diets with different dietary protein and lipid levels, exploring the mechanisms behind the differences in growth of *P. dabryanus* is critical to setting optimal nutrient requirements and achieving optimal aquaculture growth rates. In this study, *P. dabryanus* was fed a CP40EE10 diet (40% protein and 10% lipid) and reached the highest WGR and SGR. Hence, compared with other combinations, the CP40EE10 diet was the optimal dietary protein and lipid balance for *P. dabryanus* based on growth performance. LC-MS/MS-based metabolomic and RNA-seq-based transcriptomic analyses were performed to analyze the effects of dietary protein and lipids and explore the underlying mechanisms of the *P. dabryanus* fed with CP40EE10 (optimal diet), CP30EE6 (low-protein/low-lipid diet), and CP30EE14 (low-protein/high-lipid diet).

During the in-depth analysis of the DEGs, an essential functional gene, the arginase gene (*ARG*), was identified. *ARG* regulates L-arginine to produce ornithine and urea. Ornithine is a precursor of polyamines, a vital substance for regulating cell growth and promoting cell proliferation (41). As an intermediate of the urea cycle, L-arginine can alleviate ammonia poisoning with the urea cycle and avoid metabolic disorders caused by excess ammonia (42). Additionally, L-arginine can be used for ribosomal synthetic proteins (41, 43). On the other hand, studies have found that dietary protein content significantly affects amino acid metabolism (44). In this study, the expression level of *ARG* was significantly higher in the CP40EE10 group than in the other groups. Consistent with this, the level of L-arginine in the CP40EE10 group was significantly higher than that in the CP30EE6 and CP30EE14 groups. Therefore, high protein intake in the CP40EE10 group might promote amino acid metabolism, primarily through the arginine metabolism pathway, to promote *P. dabryanus* growth. This result could further support the optimal growth data.

Two other genes related to glucose metabolism have been paid attention to, namely fructose 1,6-bisphosphatase (*FBP*) and glyceraldehyde-3-phosphate dehydrogenase (*GAPDH*). Glucose, the primary energy substance of most cells, can provide energy for the body to maintain normal physiological activities through glycolysis/gluconeogenesis (16). *FBP* and *GAPDH* are essential regulators of glycolysis/gluconeogenesis, and their reduction affects energy production (45). Therefore, different gluconeogenic enzyme activities in animal tissues will lead to different growth rates, which should be the expected result. Likewise, animals fed optimal dietary proteins and lipids exhibit the high activity of enzymes related to glucose metabolism and considerable growth potential (46). In this study, the expression of genes related to glucose metabolism in the high-protein group (CP40EE10 group) was higher than in the low-protein groups (CP30EE6 and CP30EE14). This result was similar to results showing that liver gluconeogenic enzyme activity increased when rainbow trout were fed a high-protein diet (47). This can also support the result that the growth of *P. dabryanus* fed an optimal diet was better than that of a low-protein/low-lipid or low-protein/high-lipid diet. This result resembles that of the tilapia (*Oreochromis niloticus*) trial (16).

Lipids are essential macronutrients for regulating animal growth, reproduction, health, and body functions. It has been reported that glycerophosphocholine is reduced along with a decrease in compensatory liver ability (48). PC accounts for 40–50% of total cell phospholipids, and it can control lipid metabolism, mainly regulating lipid, lipoprotein, and whole-body energy metabolism (49, 50). LysoPC is an essential metabolite produced by many cells, widely distributed in various tissues. It can increase the penetration of ions in the membrane and change the mucosal barrier function (51, 52). The study found that LysoPC can promote growth and lower lipid accumulation in juvenile turbots (53). In mammals, the mTOR signaling pathway was found to play an essential role in regulating lipid metabolism gene expression. mTORC1 promotes lipid biosynthesis by being a sterol regulatory element binding protein (SREBP) (54), which is an essential class of transcription factors involved in lipid synthesis (55). mTORC1 also promotes PPARγ activity and adipogenesis (56) and regulates PPARα activity and hepatic ketogenesis (54).

In this study, the PC, LysoPC, glycerophosphocholine, phosphocholine, and PE expressions were significantly upregulated in the CP40EE10 group. Notably, the expression of these metabolite-regulating genes, such as *SPLA2*, *ETNPPL*, *pmtA*, and *mTORC1*, also increased in the CP40EE10 group. These results showed that the CP40EE10 group had better lipid metabolism ability. Similar results were observed in seabass, large yellow croakers, and grass carps (57–59). This indicates that appropriate dietary lipid levels facilitate lipid metabolism, thus promoting fish growth.

In addition to the above metabolic pathways with significant differences, there were other KEGG-enriched pathways with significant differences among the three groups. Compared with the CP30EE6 group, the protein or fat digestion and absorption, and pancreatic secretion in the CP40EE10 group were also significantly enriched, which was mainly due to the downregulated expression of *amyA*, *SPLA*2, *CPA/B*, *LIPF*, and other genes involved in the above pathways in the liver of the CP30EE6 group, indicating that low-protein/low-lipid diet could not provide enough protein and lipid to meet individual nutritional needs, resulting in slow growth. Compared with the CP30EE14 group, the PPAR signaling pathway was significantly enriched in the CP40EE10 group. *PPARα*, *ME*1, and *LPL* were all regulatory genes in this pathway. *PPARα* plays a vital role in fatty acid beta-oxidation, and the activation of the PPARα signaling pathway has a noticeable hypolipidemic effect (60). The ME1 protein is a part of the tricarboxylic acid shuttle and can be used in Fatty acid biosynthesis and many other metabolic processes (61). LPL is a rate-limiting enzyme that decomposes chylomicrons in circulating lipoprotein and significantly low-density lipoprotein triglycerides and releases FAs and glycerol (45, 57).

A high-fat diet provides more triglycerides, causing an increase in LPL enzyme activity and promoting lipolysis. From a nutritional point of view, it provides energy for the body and releases fatty acids to meet the fatty acid requirements of fish (37). In this study, the expression of *LPL* in the CP40EE10 group was higher than in the high-fat diet CP30EE14 group, which may be because its matched protein-to-lipid ratio can promote lipid metabolism and utilization. However, in the present study, the gene expression of *PPARα*, *ME*1, and *LPL* in the CP30EE14 group was significantly lower than that in the CP40EE10 group, indicating that *P. dabryanus* in the CP30EE14 group could not fully utilize dietary lipids, which may lead to impaired fat metabolism inhibiting fish growth.

## Conclusion

In conclusion, the CP40EE10 (CP: 40%, EE: 10%) diet achieved the best growth performance of *P.* dabryanus in this study. Integrated metabolomic and transcriptomic analyses were applied to investigate the changes in metabolites and genes between the CP40EE10, CP30EE6, and CP30EE14 groups. Specific metabolic pathways were identified, and the loach individuals with fast growth presented active protein and lipid metabolisms, robust signal transduction systems. These characteristics may be essential for WGR in *P. dabryanus*. The results of this study enhance our understanding of the effects of dietary protein and lipid levels on the growth of *P. dabryanus*.

## Data availability statement

The data presented in the study are deposited in the NCBI Sequence Read Archive (SRA, http://www.ncbi.nlm.nih.gov/Traces/sra) repository, accession number SRP384751, Bioproject ID: PRJNA855358.

## Ethics statement

The animal study was reviewed and approved by China Law for Animal Health Protection and Instructions (Ethics approval No. SCXK (YU2005-0001)).

## Author contributions

WZR: Visualization Preparation, Conceptualization, Methodology; LSY: Formal analysis, Data curation, Writing-Original draft preparation; ZQB: Conceptualization, Methodology, Review & Editing, Supervision; LYA: Formal analysis, Investigation, Resources; ZYZ, HHB and YCQ: Writing - Review & Editing Preparation. All authors contributed to the article and approved the submitted version.
