# Supplementary material for: Metabolomic and transcriptomic profiling reveals the effect of dietary protein and lipid levels on growth performance in loach (Paramisgurnus dabryanus)
Source: Front Immunol. 2023 Aug 1;14:1236812. doi: 10.3389/fimmu.2023.1236812 (PMC10431964; doi:10.3389/fimmu.2023.1236812)
Supplement: Supplementary file 1 [file DataSheet_1.docx]

## Supplementary Tables

**Table S 1 Splice result statistics**

| Term | All | >=500bp | >=1000bp | N50 | Total_Length | Max_Length | Min_Length | Average_Length |
| --- | --- | --- | --- | --- | --- | --- | --- | --- |
| Unigene | 57001 | 34789 | 18888 | 1744 | 62346987 | 18064 | 301 | 1093.79 |

**Table S 2 Comparison results of Reads and Unigene**

| Sample | Total reads | Total mapped reads | Mutiple mapped reads | Unique mapped reads | Reads mapped in proper pairs |
| --- | --- | --- | --- | --- | --- |
| CP30EE6_1 | 48912372(100.00%) | 46219588(94.49%) | 26586287(54.35%) | 19633301(40.14%) | 43496048(88.93%) |
| CP30EE6_2 | 49445544(100.00%) | 46052075(93.14%) | 23772903(48.08%) | 22279172(45.06%) | 42958506(86.88%) |
| CP30EE6_3 | 48542100(100.00%) | 46280405(95.34%) | 30399533(62.63%) | 15880872(32.72%) | 43689796(90.00%) |
| CP30EE6_4 | 48557594(100.00%) | 45478881(93.66%) | 25486664(52.49%) | 19992217(41.17%) | 42429862(87.38%) |
| CP30EE14_1 | 47898950(100.00%) | 44973612(93.89%) | 25806080(53.88%) | 19167532(40.02%) | 42001326(87.69%) |
| CP30EE14_2 | 47343402(100.00%) | 44557044(94.11%) | 26616698(56.22%) | 17940346(37.89%) | 41614122(87.90%) |
| CP30EE14_3 | 49771594(100.00%) | 46786018(94.00%) | 27678761(55.61%) | 19107257(38.39%) | 43822282(88.05%) |
| CP30EE14_4 | 48112142(100.00%) | 45648195(94.88%) | 29104876(60.49%) | 16543319(34.38%) | 42495130(88.33%) |
| CP40EE10_1 | 49430526(100.00%) | 46931978(94.95%) | 31170099(63.06%) | 15761879(31.89%) | 43863358(88.74%) |
| CP40EE10_2 | 48506672(100.00%) | 45823419(94.47%) | 29174585(60.15%) | 16648834(34.32%) | 42831782(88.30%) |
| CP40EE10_3 | 50190664(100.00%) | 47422977(94.49%) | 30603199(60.97%) | 16819778(33.51%) | 44433898(88.53%) |
| CP40EE10_4 | 46761518(100.00%) | 44215186(94.55%) | 28847964(61.69%) | 15367222(32.86%) | 41328268(88.38%) |

**Table S 3** **Description of up-regulated genes in metabolism-related pathways of CP40EE10 vs CP30EE6 and CP40EE10 vs CP30EE14**

| Group | Gene name | Gene description | Pathway definition |
| --- | --- | --- | --- |
| CP40EE10 vs CP30EE6 | SPLA2 | secretory phospholipase A2 | Linoleic acid metabolism  alpha-Linolenic acid metabolism  Arachidonic acid metabolism  Ether lipid metabolism |
|  |  | | |
|  | ALDH | aldehyde dehydrogenase (NAD+) | Glycolysis/Gluconeogenesis  Arginine and proline metabolism |
|  |  | | |
|  | FBP | fructose-1,6-bisphosphatase 1 | Glycolysis/Gluconeogenesis  Pentose phosphate pathway |
|  | ALDO | fructose-bisphosphate aldolase, class I |  |
|  |  | | |
|  | GAPDH | glyceraldehyde 3-phosphate dehydrogenase (phosphorylating) | Glycolysis/Gluconeogenesis |
|  | ENO | enolase |  |
|  |  | | |
|  | proA/proB | glutamate 5-kinase/ glutamate-5-semialdehyde dehydrogenase | Arginine and proline metabolism |
|  | PRODH2 | hydroxyproline dehydrogenase |  |
|  | speD, AMD | S-adenosylmethionine decarboxylase |  |
|  |  | | |
|  | GPX | glutathione peroxidase | Arachidonic acid metabolism |
|  | ENPP2 | ectonucleotide pyrophosphatase/phosphodiesterase family member 2 | Ether lipid metabolism |
| CP40EE10 vs CP30EE14 | GOT1 | aspartate aminotransferase, cytoplasmic | Phenylalanine metabolism  Phenylalanine, tyrosine and tryptophan biosynthesis  Cysteine and methionine metabolism  Tyrosine metabolism  Alanine, aspartate and glutamate metabolism  Arginine and proline metabolism  Arginine biosynthesis |
|  |  | | |
|  | TAT | tyrosine aminotransferase | Phenylalanine metabolism  Phenylalanine, tyrosine and tryptophan biosynthesis  Cysteine and methionine metabolism  Tyrosine metabolism  Ubiquinone and other terpenoid-quinone biosynthesis |
|  |  | | |
|  | HPD | 4-hydroxyphenylpyruvate dioxygenase | Phenylalanine metabolism  Tyrosine metabolism  Ubiquinone and other terpenoid-quinone biosynthesis |
|  |  | | |
|  | PAH | phenylalanine-4-hydroxylase | Phenylalanine metabolism  Phenylalanine, tyrosine and tryptophan biosynthesis |
|  |  | | |
|  | PCK | phosphoenolpyruvate carboxykinase (GTP) | Glycolysis / Gluconeogenesis  Pyruvate metabolism |
|  |  | | |
|  | LDH | L-lactate dehydrogenase | Glycolysis / Gluconeogenesis  Cysteine and methionine metabolism  Pyruvate metabolism |
|  |  | | |
|  | ACS | acetyl-CoA synthetase | Glycolysis / Gluconeogenesis  Glyoxylate and dicarboxylate metabolism  Pyruvate metabolism |
|  |  | | |
|  | MDH | malate dehydrogenase | Glyoxylate and dicarboxylate metabolism  Cysteine and methionine metabolism  Pyruvate metabolism |
|  |  | | |
|  | AGXT | alanine / serine-glyoxylate transaminase  serine-pyruvate transaminase | Glyoxylate and dicarboxylate metabolism  Alanine, aspartate and glutamate metabolism  Glycine, serine and threonine metabolism |
|  |  | | |
|  | SDS, SDH, CHA1 | L-serine/L-threonine ammonia-lyase | Cysteine and methionine metabolism  Glycine, serine and threonine metabolism |
|  |  | | |
|  | LPL | lipoprotein lipase | Glycerolipid metabolism  Cholesterol metabolism |
|  |  | | |
|  | GLUD1-2 | Glutamate dehydrogenase (NAD(P)+) | Alanine, aspartate and glutamate metabolism  Arginine biosynthesis  Nitrogen metabolism |
|  |  | | |
|  | ARG | arginase | Arginine and proline metabolism  Arginine biosynthesis |
|  |  | | |
|  | G6PC | glucose-6-phosphatase | Glycolysis / Gluconeogenesis |
|  | FBP | fructose-1,6-bisphosphatase |  |
|  | ALDO | fructose-bisphosphate aldolase, class Ⅰ |  |
|  | GAPDH | glyceraldehyde 3-phosphate dehydrogenase (phosphorylating) |  |
|  |  | | |
|  | CDO1 | cysteine dioxygenase | Cysteine and methionine metabolism |
|  | iLVE | branched-chain amino acid aminotransferase |  |
|  | achY | adenosyl homocysteinase |  |
|  | MAT | S-adenosylmethionine synthetase |  |
|  |  | | |
|  | proB | glutamate 5-kinase | Arginine and proline metabolism |
|  | proA | glutamate-5-semialdehyde dehydrogenase |  |
|  | PRODH | proline dehydrogenase |  |
|  | OAT | ornithine--oxo-acid transaminase |  |
|  | speG, SAT | diamine N-acetyltransferase |  |
|  | speD, AMD1 | S-adenosylmethionine decarboxylase |  |
|  |  | | |
|  | maeB | malate dehydrogenase (oxaloacetate-decarboxylating) (NADP+) | Pyruvate metabolism |
|  | ACACA | acetyl-CoA carboxylase / biotin carboxylase 1 |  |
|  |  | | |
|  | GCH1 | GTP cyclohydrolase ⅠA | Folate biosynthesis |
|  | PAH, phhA | phenylalanine-4-hydroxylase |  |
|  | PCBD, phhB | 4a-hydroxytetrahydrobiopterin dehydratase |  |
|  |  | | |
|  | HGD | homogentisate 1,2-dioxygenase | Tyrosine metabolism |
|  | AOX | aldehyde oxidase |  |
|  |  | | |
|  | TGL2, lip | triacylglycerol lipase | Glycerolipid metabolism |
|  | PLPP | phosphatidate phosphatase |  |
|  |  | | |
|  | ABCA1 | ATP-binding cassette, subfamily A(ABC1), member 1 | Cholesterol metabolism |
|  | CYP7A1 | cholesterol 7alpha-monooxygenase |  |
|  | LIPG | endothelial lipase |  |
|  | LDLR | low-density lipoprotein receptor |  |
|  |  | | |
|  | SARDH | sarcosine dehydrogenase | Glycine, serine and threonine metabolism |
|  | CA | carbonic anhydrase | Nitrogen metabolism |

**Table S 4 Description of up-regulated genes in non-metabolically related pathways of CP40EE10 vs CP30EE6 and CP40EE10 vs CP30EE14**

| Group | Gene name | Gene description | Pathway definition |
| --- | --- | --- | --- |
| CP40EE10 vs CP30EE6 | amyA | alpha-amylase | Pancreatic secretion |
|  | PLA2G/SPLA2 | secretory phospholipase A2 |  |
|  | CCKAR | cholecystokinin A receptor |  |
|  | PMCA/ATP2B | P-type Ca2+ transporter type 2B |  |
|  | CETR | cystic fibrosis transmembrane conductance regulator |  |
|  |  | | |
|  | PRSS_1_2_3 | trypsin | Pancreatic secretion  Protein digestion and absorption |
|  | CTRB | chymotrypsin |  |
|  | CTRL | chymotrypsin-like protease |  |
|  | CELA | pancreatic elastase II |  |
|  | CPA | carboxypeptidase A1 |  |
|  | CPB | carboxypeptidase B |  |
|  | CEL | bile salt-stimulated lipase |  |
|  |  | | |
|  | LIPF | gastric triacylglycerol lipase | Fat digestion and absorption |
|  | SCARB1 | scavenger receptor class B, member 1 |  |
|  | NPC1L1 | Niemann-Pick C1-like protein 1 |  |
| CP40EE10 vs CP30EE14 | ATPA1 | sodium/potassium-transporting ATPase subunit alpha | Proximal tubule bicarbonate reclamation |
|  | CA2 | carbonic anhydrase 2 |  |
|  | GLUD1_2/gdhA | glutamate dehydrogenase (NAD(P)+) |  |
|  | MDH1 | malate dehydrogenase |  |
|  | PCK | phosphoenolpyruvate carboxykinase (GTP) |  |
|  |  | | |
|  | PPARα | peroxisome proliferator-activated receptor alpha | PPAR signaling pathway |
|  | ME1 | malate dehydrogenase (oxaloacetate-decarboxylating) (NADP+) |  |
|  | FADS2 | acyl-CoA 6-desaturase (Delta-6 desaturase) |  |
|  | CYP7A1 | cholesterol 7alpha-monooxygenase |  |
|  | LPL | lipoprotein lipase |  |
|  | ACS | long-chain acyl-CoA synthetase |  |
|  | UBC | ubiquitin C |  |
|  | PCK | phosphoenolpyruvate carboxykinase (GTP) |  |

## Supplementary Figures


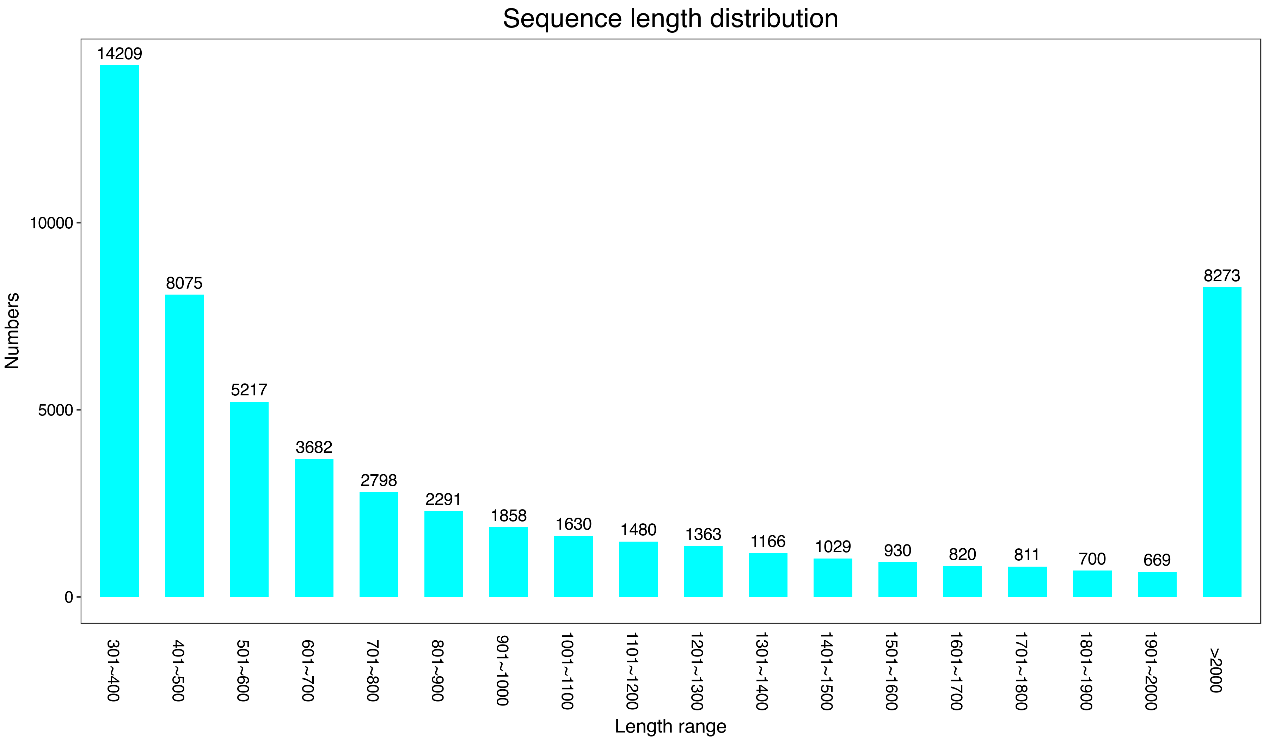
A


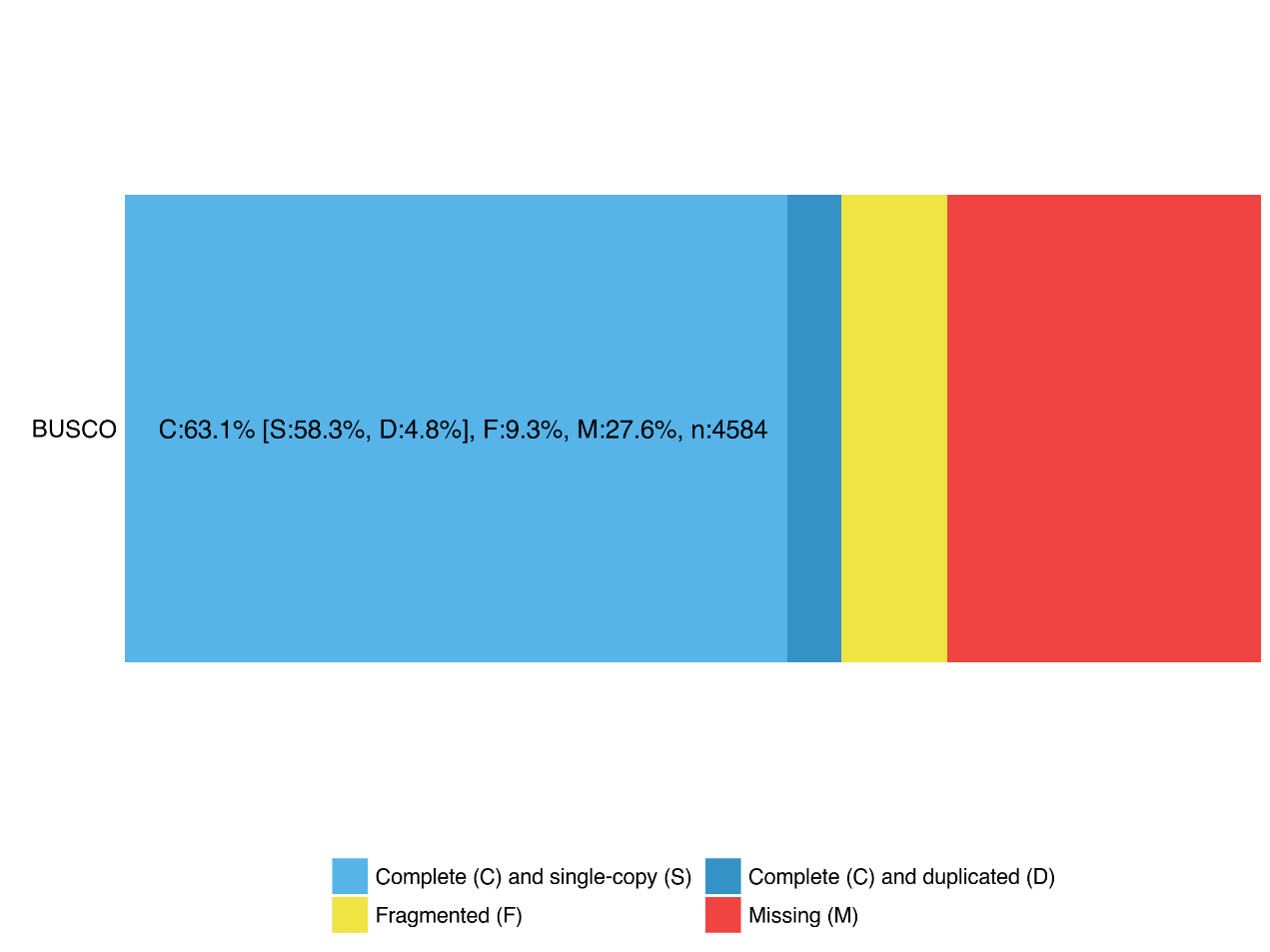
B

**Fig. S 1 Unigene length distribution (A) and BUSCO integrity assessment statistics chart (B)**
